# Biopolymer-Based Nanogel Approach in Drug Delivery: Basic Concept and Current Developments

**DOI:** 10.3390/pharmaceutics15061644

**Published:** 2023-06-02

**Authors:** Ebru Altuntaş, Burcu Özkan, Sevgi Güngör, Yıldız Özsoy

**Affiliations:** 1 Faculty of Pharmacy, Department of Pharmaceutical Technology, Istanbul University, 34116 Istanbul, Türkiye; ebru.altuntas@istanbul.edu.tr (E.A.); sgungor@istanbul.edu.tr (S.G.); 2 Graduate School of Natural and Applied Science, Yildiz Technical University, 34220 Istanbul, Türkiye; burcu_ozkan93@hotmail.com

**Keywords:** biopolymers, nanogels, drug delivery, polysaccharide-based nanogels, protein-based nanogels, nanotechnology

## Abstract

Due to their increased surface area, extent of swelling and active substance-loading capacity and flexibility, nanogels made from natural and synthetic polymers have gained significant interest in scientific and industrial areas. In particular, the customized design and implementation of nontoxic, biocompatible, and biodegradable micro/nano carriers makes their usage very feasible for a range of biomedical applications, including drug delivery, tissue engineering, and bioimaging. The design and application methodologies of nanogels are outlined in this review. Additionally, the most recent advancements in nanogel biomedical applications are discussed, with particular emphasis on applications for the delivery of drugs and biomolecules.

## 1. Biopolymer-Based Microgels/Nanogels as a Drug Delivery System

Researchers are constantly exploring new materials with enhanced properties that can be employed in a number of biomedical applications, including as drug delivery systems, prosthetic devices, theranostics, drug targeting, magnetic resonance imaging, and tissue engineering scaffolds [1]. Through the creation of materials at the nanoscale level, the development of nanotechnology altered various medical processes and technologies. Due to their improved characteristics over their bulk counterparts, nanoscale materials are important in the field of drug delivery. Recent research demonstrated the significance of the nanoscale size range in a variety of drug delivery methods, including hydrogels, which sparked the creation of micro- (microgels) and nanoscale hydrogels (nanogels) [2,3]. Microgels, also referred to as nanogels, are hydrogel particles sized in the submicron range [4,5]. The 3D cross-linked hydrogel nanoparticles known as nanogels have grown in attraction as nanoparticulate drug delivery systems [6]. Three-dimensional hydrogel particles with submicron particle sizes are used in nanogel delivery systems. Nanogels are produced in aqueous solutions by combining hydrophilic, hydrophobic, or amphiphilic polymers chemically or physically (noncovalent attractive forces such as hydrophilic–hydrophilic, hydrophobic–hydrophobic, ionic contacts, and/or hydrogen bonding) [7,8,9,10]. Nanogels have the ability to absorb liquid while maintaining their structural integrity thanks to polymer internal crosslinking [11].

Nanogels can be produced using polymers that are synthetic, natural, or a combination of both and depending on the methods of synthesis applied, nanogels can be formed into various shapes such as core–shell structures, spherical particles, or core–shell–corona structures [12]. Proteins and polysaccharides that are chosen for their biodegradability and low immunogenicity may also be included in nanogel-forming components. These are designed to be very effective at enhancing the drug payload in the targeted area and reducing the tendency that the loaded bioactives will leak from all other nanocarriers [10]. Size, biocompatibility, charge, degradability, porosity, and other characteristics of nanogels can be successfully modified by changing their chemical composition [8,9,13,14]. Because of the extremely high interfacial area per unit mass, both micro- and nanoscale hydrogels respond to various environments more quickly than their bulk counterparts and have a rapid exchange rate [15,16]. 

Nanogel solutions behave like a dilute colloidal system at low concentration and the size of these uniformly dispersed nanogel particles and the local concentration of the polymer chains that are crosslinked in each particle can be altered throughout the polymerization process by adjusting crosslink density and surfactant concentration [17,18]. Their hybrid features result from combining the characteristics of a gel and a colloid, such as small size, high surface-to-volume ratio, and micro-heterogeneous structure [19]. Given their advantages over different systems including their hydrophilicity, biocompatibility, higher colloidal stability, degradability, adjustable size, three-dimensional structure, and ease of production, nanogels are regarded as the next generation of drug delivery technologies [20,21]. Furthermore, nanogels have particular benefits over different kinds of nanomaterials in the biomedical area owing to their capacity to respond quickly to environmental changes such as temperature, ionic strength, light, and pH [9,22]. 

The presence of COOH, OH, NH_2_, CONH, SO_2_H, CONH_2_, etc., in nanogels contributed to their ability to absorb high quantities of biological fluids and water [23]. Hydrophilic polymers can exhibit hydration of up to 90% in contrast to hydrophobic polymers, which can only show a 5–10% hydration level [24]. Critical parameters such as shape, size, swelling intensity, and chemical and topological composition can be adjusted to obtain the special features of nanogels [25].

## 2. Advantages of Biopolymer-Based Nanogels over Other Drug Delivery Systems

Pharmaceutical nanotechnology focuses on the development of therapeutically effective, biocompatible, and biodegradable nanoparticulate systems for enhanced drug bioavailability, targeted drug administration, and stability against chemical/enzymatic degradation [26]. Nanogels are regarded as a potential drug delivery system because of their beneficial features that combine those of hydrogels and nanoparticles. Nanogels have been thoroughly studied to deliver a variety of bioactive substances, including proteins, drugs, and vaccines [8,27,28,29].

Nanogels are considered superior to alternative drug delivery techniques for a variety of reasons. They are relatively more effective and much safer delivery systems for both hydrophobic and hydrophilic drugs due to their chemical components and formulation characteristics. They have made possible the expansion of functionalized nanoparticles, which serve as drug carriers and enable the controlled release of drugs and other active substances at specified sites [30].

Nanogels exhibit great properties including ease of synthesis, high stability and loading capacity, size control, effective drug encapsulation, controlled and prolonged drug release, good hydrophilicity and good permeation capability because of smaller size, solubility, both active and passive targeting, viscoelasticity, low toxicity electromobility, biocompatibility and biodegradability, as well as high ionic strength response, biomolecule identification, pH, temperature light, magnetic field, and different environmental factors [8,9,13,14,31,32,33].

## 3. Synthesis of Nanogels

Universally, the current approaches towards preparation of nanogels include four different procedures, listed below.

### 3.1. Physical Self-Assembling of Interacting Polymer Chains

The controlled aggregation of the hydrophilic polymers employing noncovalent or lower interactions including hydrophilic–hydrophilic, hydrophobic–hydrophobic, van der Waals forces, ionic interactions, and hydrogen bonding causes the physical self-assembly of the interactive polymers [34]. In the case of nanogel formation, controlled aggregation of amphiphilic or hydrophilic polymers that can interact through hydrophobic, electrostatic, and/or hydrogen bonding interactions is typically involved in physical self-assembly. The formation of these nanogels typically takes place in an aqueous solution under mild circumstances. The proper selection of polymer content, amphiphilic nature, functional groups, pH, ionic strength, and temperature determines the size of nanogels [35]. On the other hand, the degree of crosslinking is another important factor to be considered on nanogel parameters, such as the nanoparticle size, swelling degree, drug release profile, and overall therapeutic response [36]. Physical crosslinking produces nanogels in a matter of minutes, resulting in the association and complex formation of the necessary polymeric chains [37]. Due to its simplicity, this method is the most commonly used process in nanogel manufacture. It comprises blending the polymer as a carrier and the drug to be loaded [38,39]. Because of the high hydrophilic interactions involved, hydrophilic polymers are the most preferred materials in this method of nanogel formation because they provide the most stable crosslinked nanogels [36].

Physical self-assembly, in particular, can be used to manufacture polysaccharide-based nanogels. Polysaccharides are hydrophilic polymers that have hydrophobic groups attached to them. In the presence of such modified polymers, the hydrophobic moieties interact with one another, increasing the production of nanogels favorable for the transport of active compounds [40]. Li et al. coupled ovomucin with chitooligosaccharide by self-assembly, followed by condensation of complexes triggered using glycerol solution to obtain an ecofriendly nanogel containing curcumin [41]. In another study, Atallah et al. described the creation of self-assembled nanogels using hydrophilic biocompatible proteins, lactoferrin, and polysaccharide carboxy methyl cellulose for the dual delivery of the antimetabolite pemetrexed and the natural polyphenol honokiol [42].

When compared to covalent bonds, noncovalent interactions between polymer chains are significantly weaker. As a result, stable nanogels of controllable size might be more difficult to manufacture using this method. Nevertheless, physically crosslinked nanogels have several advantages against chemically crosslinked nanogels, including the absence of a potentially toxic crosslinker and/or catalyst [5]. 

### 3.2. Chemical Crosslinking of Preformed Polymers

Chemically crosslinked gels are distinguished by the existence of constant chemical connections that are formed by strong bonds such as covalent bonds across the gel networks, and the physicochemical characteristics of such gels may differ based on the chemical connections and functional groups [43]. 

Three phases typically comprise the gelation process: the production of aggregates by hydrophobic contact; the strengthening of the aggregate particles via chemical crosslinking; and the improvement in deformability upon cooling as a result of the formation of numerous hydrogen bonds. For the creation of nanogels with porous or micellar network architectures, chemical crosslinking is appropriate [5]. 

Carbodiimide coupling, Michael addition reaction, and free radical polymerization are three methods for synthesizing biodegradable dextran-based microgels and hydrogels that rely on chemical crosslinking [44]. 

A further example involves the introduction of a novel, simple, and reliable technique for making tunable DNA-protein nanogels with adjustable size and density. Highly biotinylated DNA was manufactured for this use using a polymerase chain reaction as a soft biopolymeric backbone that can be effectively crosslinked using streptavidin–biotin binding [45].

### 3.3. Polymerization of Monomers in a Homogeneous Phase or a Micro- and/or Nanoheterogeneous Phase

Emulsion polymerization and inverse emulsion polymerization are the two categories of polymerization that are appropriate for the development of nanogels.

In the emulsion polymerization method, free radical polymerization is used as the process mechanism, a common technique for creating nanogel systems. In this procedure, radical addition and polymerization take place in a heterogeneous system. The subsequent step is the emulsification of a monomer (hydrophobic in nature) in oil and water emulsion. Any kind of water- or oil-soluble free radical initiator might be used to attempt to initiate the polymerization reaction, which would result in polymer dispersion. This procedure uses polymers, which may be either natural or artificial [36]. 

In order to prepare carmofur-loaded nanogels based on biocompatible and temperature/pH-sensitive monomers such as polyethylene glycol diacrylate (PEGDA), N-vinylcaprolactam (NVCL), and 2-(dimethylamino) ethyl methacrylate (DMAEMA), a straightforward and efficient strategy of one-pot laser-induced emulsion polymerization at 532 nm was recently developed due to their ideal size, excellent hydrophilicity, good biocompatibility, and sensitivity to particular stimulation, to increase the efficacy of chemotherapy [46].

In the inverse emulsion polymerization method, inverse water in oil nanoemulsion is employed as a polymerization medium for monomers. In fact, after the addition of particular comonomers, which act as bifunctional crosslinkers, stable nanogels are achieved. Aqueous suspensions or oil-in-water nanoemulsions can also be used to perform the polymerization that results in nanogels. In other instances, the polymerization might begin in a homogenous aqueous solution, which transforms into a milky suspension during polymerization and contains the developing nanogel. The finished product is then freeze dried to remove it from suspension [35]. 

Inverse emulsion polymerization is a particularly well-liked method for creating hydrophilic nanoparticles, hydrogels, microgels, and nanogels. Surfactants with low hydrophilic lipophilic balance values, or more hydrophobic emulsifiers, are used to emulsify the aqueous phase in oil. Steric effects result in stabilization of the system. Typically, inverse emulsions are thermodynamically unstable. In the presence of high emulsifier concentrations (>8% wt), less aqueous phase (15% *v/v*), and the addition of co-emulsifiers, thermodynamic stability can be obtained [47].

By using the inverse emulsion process, oxidized sucrose crosslinked Schizophyllan nanogel was successfully created. There was no use of an organic solvent or a hazardous crosslinker during the synthesis procedure. Fractionated coconut oil was used as the dispersion medium and oxidized sucrose as the crosslinker to create the nanogel crosslinking network [48].

### 3.4. Template-Assisted Nanofabrication

This innovative imprinting technique, also known as particle replication in non-wettable templates (PRINT), was created by DeSimone and is appropriate for the fabrication of nanogels [49]. This technique can be used to create polymeric nanoparticles with sizes ranging from tens of nanometers to several micrometers [35]. 

The liquid precursor is kept inside non-wetting molds in this top-down method. Utilizing a mold allows for precise control over particle size, shape, composition, and surface. It also aids in preventing the formation of interconnecting films between the molded items, leading to the production of monodispersed particles with good size and shape uniformity. Using UV-assisted copolymerization of different monomers, such as PEG diacrylate, PEG monoethyl ether, and monomethacrylate, monodispersed, 200 nm diameter swellable PEG-based nanoparticles were created [50]. 

## 4. Nanogels of Natural Polymers

Nanogels have a wide range of remarkable applications since they are ideal for encapsulating a variety of different bioactive substances. Nevertheless, for the efficient application of nanogels in pharmaceutical fields, biopolymers that are generally considered as safe (GRAS) are needed instead of synthetic polymers or biopolymers with a chemical modification [51]. The production of polysaccharide and protein nanogels without toxic and harmful crosslinking agents is necessary to ensure people’s safety and acceptance [52].

Various colloidal delivery systems for pharmaceutical applications can be produced using natural biopolymers, such as proteins and polysaccharides or by combining these materials to create nanogels that are biopolymer-based complexes. The next section discusses the varieties and advantages of protein- and polysaccharide-based nanogels in the pharmaceutical area.

### 4.1. Polysaccharide-Based Nanogels

#### 4.1.1. Advantages of Polysaccharide Nanogels

A family of carbohydrates known as polysaccharides has massive polymeric oligosaccharides that are created through glycosidic connections between several monosaccharide repeats [53]. 

In nature, components of plant (examples include pectin, cellulose, starch), animal (examples include chitosan, chitin, glycosaminoglycan), microbial (examples include dextran, pullulan, xanthan gum, and gellan gum), and algal origin (examples include agar, alginate, and carrageenan) are the primary sources of polysaccharides [54,55].

Homopolymers (i.e., made from the same monosaccharide repeats as glycogen, pullulan, starch, cellulose, pectin, etc.) and heteropolymers (made from distinct monosaccharide repeats as heparin, hyaluronic acid, chitosan, keratan sulfate, chondroitin sulfate, heparan sulfate, and dermatan sulfate) are two different types of polysaccharides that depend on the makeup of their monosaccharide units [56,57,58].

Thanks to their physicochemical and biological characteristics, polysaccharide-based nanoparticles are very important as a carrier of several medicinal drugs [59]. In addition to allowing specific receptor attachment or recognition, neutral coatings with low surface energies are also provided by polysaccharides, preventing nonspecific protein adsorption and providing neutral coatings [60]. Furthermore, the presence of multifunctional groups on the polysaccharide backbone, such as hydroxyl, carboxyl, and amine groups, enables chemical or enzymatic derivatization with other compounds. The ideal choices for nanogel synthesis in drug delivery systems are polysaccharides because of their high availability in nature, biocompatibility, biodegradability, nontoxicity, solubility in water, low immunogenicity, and ease of chemical or enzymatic modification [61]. They can also be extracted, refined, and processed utilizing ecofriendly, green methods [1]. The main disadvantages of polysaccharide drug delivery may be their inherent unpredictability and challenging lab manufacturing [59].

#### 4.1.2. Natural Polysaccharides Used in Nanogels

- Chitosan

Deacetylated chitin (poly-N-acetyl glucosamine) comprising β-1,4-linked glucosamine (2-amino-2-deoxy-β-D-glucose) provides the basis for the compound chitosan, which also contains trace amounts of N-acetyl glucosamine [62]. A naturally occurring polymer, chitosan is mostly generated from the chitin in the shells of marine animals. The exoskeletons of crustaceans including shrimp, lobsters, and crabs are a source of chitin. Additionally, fungus and yeast are organisms that produce chitosan [63,64,65]. 

In 1948, a deacetylation reaction using heat and strong alkali resulted in the first conversion of chitin to chitosan [66]. Chitosan typically has a pKa value between 6.3 and 6.6. Formic, acetic, tartaric, and citric acids are only a few of the organic acids in which chitosan is soluble. The substance clumps at neutral pH levels and is insoluble in water and alkaline solutions [63].

Chitosan is the second most common polysaccharide after cellulose. It is employed in drug delivery applications due to its reactive functional groups, biocompatibility, biodegradability, ability to form gels, nontoxicity, and high positive charge density [67,68]. Additionally, it contains mucoadhesive, antibacterial, and antifungal characteristics, as well as functionality that improves permeation [69]. Furthermore, chitosan controls the expression of growth factors, as demonstrated in a study using a mouse burn wound model. In this study, TGF-b1 expression and collagen production were elevated for the first three days, promoting tissue regeneration, and decreased at day seven, preventing collagen-excessive production and scarring [70,71].

Due to their high payload capacity, low toxicity, and ability to modify release profiles, chitosan microgels and nanogels are frequently used in pharmaceutical delivery systems [65]. They also adhere well to mucosal surfaces and have a propensity to increase epithelial permeation by temporarily opening hard epithelial components [72]. Chitosan interacts with anions, other polyelectrolytes (such as alginate and carrageenan), fatty acids, and proteins since it is a polycation by nature. Additionally, differences in intrinsic viscosity can be seen in the medium, which depend on its pH and ionic strength [73]. It is important to note that chitosan networks are typically used to obtain pH-sensitive compounds [1]. In the literature, covalent crosslinking of carboxymethyl starch and chitosan hydrochloride successfully produced nanogels as promising delivery vehicles for curcumin. The nanogels exhibited great pH sensitivity and high curcumin encapsulation efficiency (89.49–94.01%). In simulated gastrointestinal circumstances, curcumin-encapsulated in nanogels displayed a sustained release profile as opposed to free curcumin [74]. 

**Figure 1:**
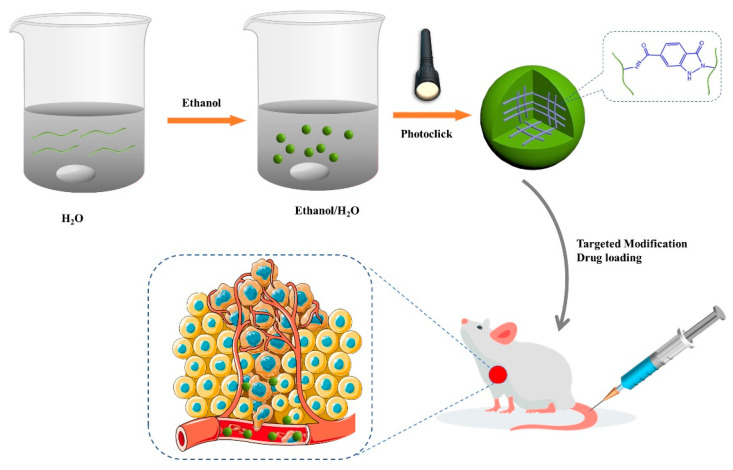
Schematic illustration of o-nitro-benzyl alcohol-modified carboxymethyl chitosan (CMC-NBA) nanogels prepared using catalyst-free photo click reaction and their tumor-targeting drug delivery in vivo. Reprinted from [75], Copyright (2022), with permission from Elsevier.

A straightforward method for producing chitosan-based nanogels was also reported [75]. It used photocrosslinking, poorly solvent-induced nanoaggregates without the use of an emulsifier, catalyst, or external crosslinker. In this study, carboxymethyl chitosan that had been treated with o-nitro-benzyl alcohol was created, and it then self-crosslinked into nanogels in a mixture of ethanol and water under 365 nm light irradiation due to primary amine and o-nitro-benzyl alcohol cyclization caused by ultraviolet light (Figure 1). The nanogels and lactobionic acid-decorated nanogels showed good stability and a consistent diameter of about 200 nm. The nanogels, in particular, showed a high loading percentage of around 28%. The findings indicated that, in mildly acidic circumstances, doxorubicin-loaded nanogels demonstrated fast drug release. The lactobionic acid-decorated nanogels were found to be more effective than the nanogels at increasing cellular absorption, improving cytotoxicity in tumor cells, and enhancing growth inhibition in vivo, according to both cell and animal tests. So, there is a great deal of potential for doxorubicin delivery with these photocrosslinked nanogels.

**Figure 2:**
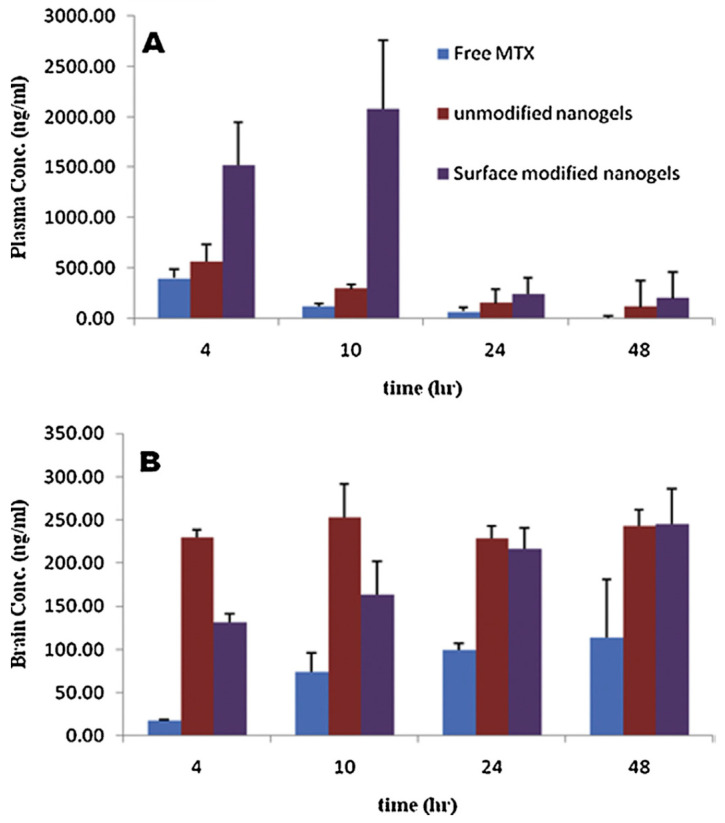
Following a 25 mg/kg IV dosage of free solution, loaded using unmodified nanogels, and loaded using surface-modified nanogels, time courses of methotrexate concentrations (ng/mL) in the brain (**B**) and plasma (**A**) in male Sprague–Dawley rats. Reprinted from [76], Copyright (2013), with permission from Elsevier.

Methotrexate-loaded nanogels were synthesized by Azadi et al. using the ionic gelation technique, and after being characterized in vitro, the transport of the nanogels across the blood–brain barrier was examined in vivo in healthy animals [76]. Consequently, after intravenous administration of surface-modified and unmodified nanogels in comparison to the free drug, all at the same dose of 25 mg/kg, the nanogel formulations led to greater methotrexate concentrations in the brain than with the free drug (in some cases, more than 10-fold); however, there were no substantial differences between the surface-modified and unmodified nanogels in any of the time points evaluated (Figure 2).

- Dextran

Another naturally occurring biopolymer in the structure of carbohydrates is dextran. Because of the presence of hydroxyl groups and a biocompatible polysaccharide polymer, the molecular structure is very hydrophilic. It is often synthesized using enzymatic conversion and is composed of linear chains of d-glucopyranose residues that are 1,6-linked [77]. 

The therapeutic application of dextran-based materials as a blood plasma volume enhancer and anticoagulant therapy is supported by their biocompatibility, high degree of hydrophilicity, and minimal protein adsorption. Dextran also serves as an adjuvant, stabilizer, emulsifier, and carrier in food, pharmaceutical, and chemical applications [78]. Dextran, like other polysaccharides, has a number of possibilities for derivatization with other compounds to create dextran-based nanoparticles [79,80,81].

In a work by Zhang et al., soybean protein isolate (SPI)-dextran conjugate-based nanogels were manufactured using the Maillard reaction together with protein self-assembly [82]. For the purpose of fabricating SPI-dextran conjugate (SDC), the dextran molecular weight (40 kDa), SPI/dextran mass ratio (1:1.75), and incubation period (3.3 d) were determined. The SDC-based nanogels showed good durability against heat treatment, ionic strength, and storage and were transparent in aqueous solution. They also had a spherical core–shell structure with a Dh of 104.4 nm. The findings showed that SDC-based nanogels could be employed as desirable nanocarriers for entrapping hydrophobic bioactive chemicals. 

**Figure 3:**
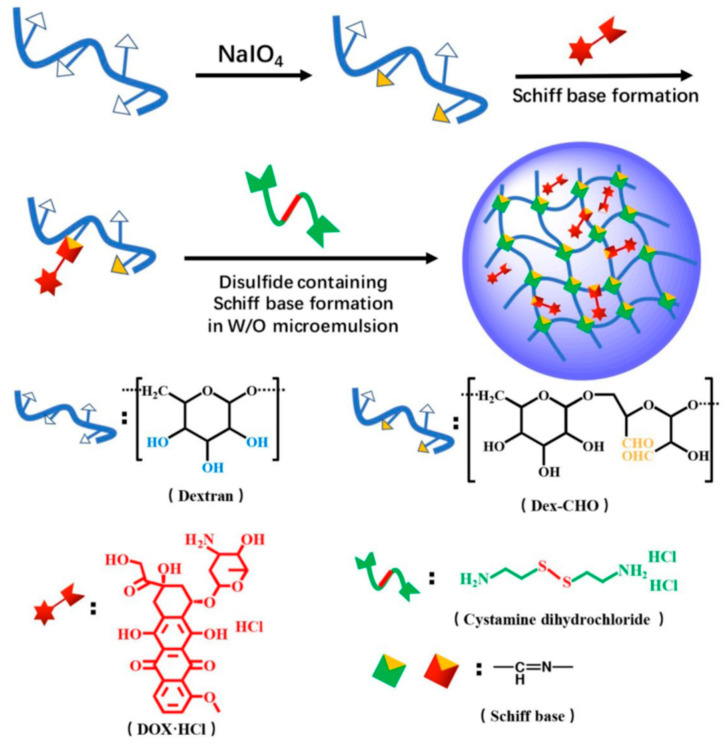
A schematic illustration for the creation of Schiff base formations incorporating disulfides in a *w*/*o* microemulsion to create a pH/reduction dual sensitive dextran nanogel [83] (reuse permitted by Elsevier).

Polymeric nanoparticles that are dual- and multi-stimuli-sensitive and can react to two or even more signals have been shown to be potential drug carriers with improved tumor accumulation and on-demand drug release patterns [83]. A simple approach based on the disulfide-containing Schiff base synthesis between polyaldehyde dextran and cystamine in a water-in-oil inverse microemulsion was used to create dextran-based nanogels (Figure 3). 

Using Schiff base linkages, doxorubicin was covalently attached into the dextran nanogels, and pH and acidic-reductive dual sensitive drug release patterns were shown. Human cancer cell line H1299 was used to determine the precise cell import of the doxorubicin-loaded nanogels. The dual-stimuli-responsive dextran-based nanogels can act as microenvironment-sensitive drug delivery vehicles for tumor therapy by taking advantage of the acidic and reductive tumor microenvironment.

Novel curcumin-based biodegradable nanogels were developed by K. Nagahama et al. [84] through the self-assembly of amphiphilic dextran–curcumin conjugates. They claimed that the synthetic nanogels considerably increase curcumin loading amounts and improve solubility of hydrophobic curcumin in water. Additionally, the in vitro cell uptake results demonstrate that HeLa cells efficiently absorbed dextran–curcumin nanogels and displayed high fluorescence suitable for live cell imaging. Dextran–curcumin nanogels may therefore hold promise for the development of innovative, highly effective curcumin-based cancer therapeutics. Dextran-based nanogels are also employed to transport proteins and siRNA in addition to chemotherapeutic medications [85,86]. 

Redox-sensitive dextran nanogels were created by Li et al. [87] in order to deliver antigens directly inside of cells (ovalbumin, OVA). Although dextran nanogels’ disulfide bonds are durable outside of cells, they weaken in dendritic cells’ cytoplasm because glutathione is present there. Additionally, OVA-conjugated nanogels demonstrate the viability of this idea for the intended intracellular antigen delivery by enhancing MHC class I antigen presentation and demonstrating intracellular release of the antigen in dendritic cells.

- Heparin

Heparin is a linear polysaccharide made up of uronic acid repeats at random intervals, one to four bonded disaccharides, and glucosamine residues. Heparin-loaded hydrogels have been investigated for their numerous applications and functionalizations, such as implantation, tissue engineering, biosensors, and drug-controlled release [88,89].

Pyranosyluronic acid and glucosamine residues make up heparin. Due to their crosslinking properties, heparin-based nanohydrogels, which are anionic in nature, have been employed for drug administration [90]. In earlier research, heparin polymers were also reported to possess anticancer activities [91].

Pyranosyluronic acid and glucosamine residues make up the chemical structure of heparin. Heparin nanohydrogels are anionic in nature, and heparin-based nanohydrogels have been used for drug delivery due to their crosslinking nature [90]. Heparin polymers have also been reported to have anticancer properties in earlier studies [91]. There have been claims that low molecular weight heparin (LH) possesses antifibrotic and anticancer activities. A low molecular weight heparin–pluronic nanogel (LHP) was made by conjugating carboxylated pluronic F127 to LH in order to increase the efficacy and reduce side effects of LH. About 33% of the intrinsic anticoagulant activity was lowered by the LHP. Aspartate transaminase, alanine transaminase, total bilirubin, and direct bilirubin levels all decreased after LHP treatment, which also stopped DMN-mediated liver weight loss. Compared to LH, LHP significantly decreased the fibrotic area. Additionally, in DMN-induced liver fibrosis, LHP potently suppressed the expression of mRNA or proteins for alpha-smooth muscle actin, collagen type I, matrix metalloproteinase-2, and tissue inhibitor of metalloproteinase-1 compared to LH. The findings suggest that LHP inhibits the TGF-/Smad pathway and eliminates the extracellular matrix to have an anti-fibrotic impact in the liver [92].

- Pectin

About one-third of the cell walls of higher plants are made up of pectin, a significant linear heteropolysaccharide [93,94]. D-galacturonic acid units linked by -(1-4) glycosidic connections make up the bulk of natural pectin [95]. Due to its inexpensive cost of manufacture and easy accessibility, pectin and its derivatives are currently being used for drug administration increasingly and more frequently, similar to other polysaccharides [96]. 

**Figure 4:**
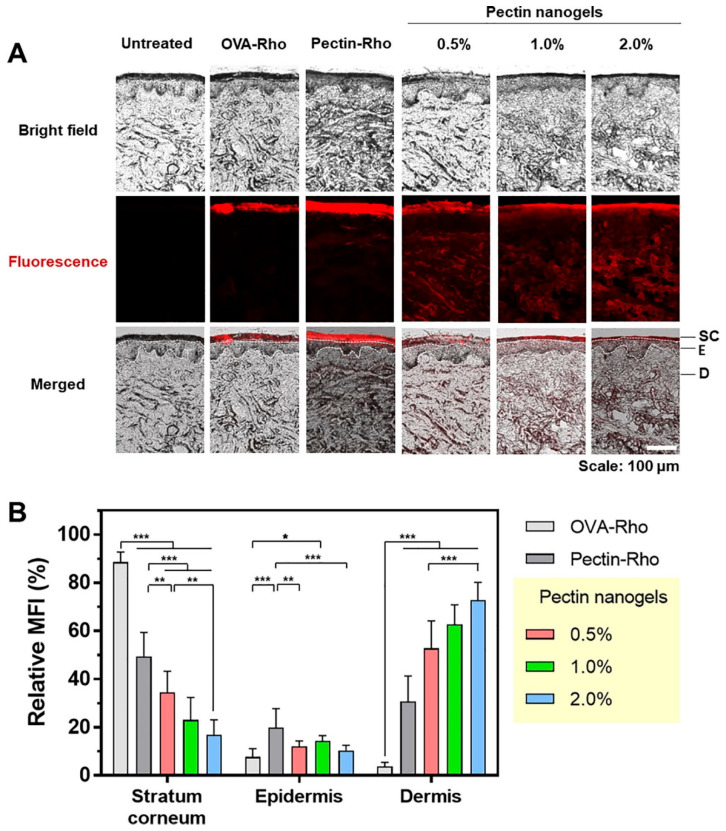
Porcine skin penetration. In Franz diffusion cells, porcine skin was coated with Rhodamine B-labeled OVA, soluble pectin, and OVA-loaded pectin nanogels of varied pectin concentrations. (**A**) Histological sections captured in bright-field and fluorescence following a 24 h incubation (SC: stratum corneum; E: epidermis; D: dermis). (**B**) Semiquantitative analysis of the fluorescence values. Every relative fluorescence intensity shows the amount of fluorescence compared to the overall amount of fluorescence (n = 8, mean ± SD) (* *p* ≤ 0.05; ** *p* ≤ 0.01; *** *p* ≤ 0.001) [97] (reuse permitted by Elsevier).

In a recent study, pectin nanogels with norbornene group-functionalized pectin, dithiol crosslinker, and thiolated OVA were created using the thiol–norbornene photo-click reaction and ultrasonication to create a novel transcutaneous antigen delivery carrier [97]. While soluble OVA did not pass through the stratum corneum layer, the OVA-loaded pectin nanogels passed and were deposited in both the epidermis and dermis (Figure 4). Dendritic cells generated from THP-1 monocytes absorbed the nanogels, which caused the overexpression of maturation markers. These findings suggest that pectin nanogels are potential transcutaneous antigen delivery vehicles.

**Figure 5:**
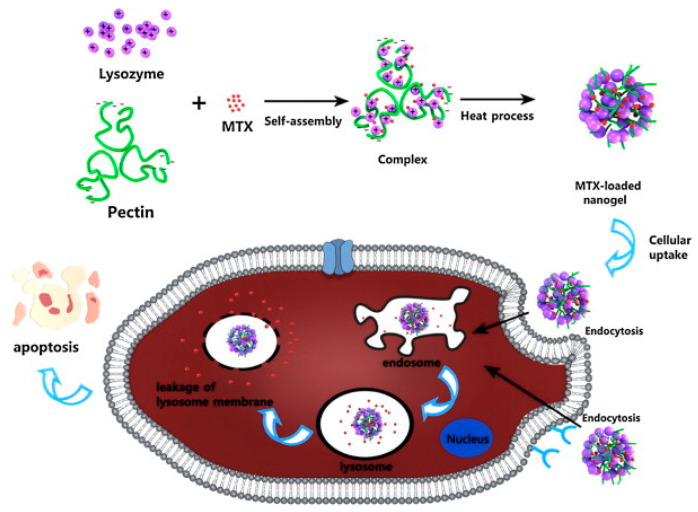
Producing MTX-loaded nanogels using lysozyme and pectin for effective intracellular MTX release, internalization by cancer cells, and induction of apoptosis [98] (reuse permitted by Elsevier).

Through self-assembly, new nanoparticles were created from lysozyme–pectin, and the resulting nanogels could be employed as a delivery system for the anticancer drug methotrexate (MTX) (Figure 5) [98]. 

The nanogels displayed negative surface charge, small particle size distribution, and spherical shapes with diameters of around 109 ± 2 nm. Furthermore, the addition of MTX did not significantly alter the particle size or shape of the nanogels. The maximum loading capacity for MTX in nanogels is 17.58 ± 0.85%. When the pH decreased from 7.4 to 5.3, MTX-loaded nanogels started to release the drug more rapidly. The MTT experiment revealed that the anticancer activity of encapsulated MTX was greater than that of free MTX. In contrast to free MTX, HepG2 cells could successfully endocytose MTX-loaded nanogels, which increased cancer cell death. It demonstrated that the nanogels had minimal toxicity and good biocompatibility. The developed nanogels held enormous promise for the creation of a novel nanocarrier for the delivery of anticancer drugs.

- Hyaluronic Acid (HA)

The extracellular tissue matrix of vertebrates contains HA, a naturally occurring linear anionic polysaccharide (glycosaminoglycan) [99]. The two disaccharide units (d-glucuronic acid and N-acetyl-d-glucosamine) that make up its chemical structure are polymerized into massive macromolecules with up to 30,000 repeating units. HA is nontoxic, nonthrombogenic, nonimmunogenic, biodegradable, biocompatible, and noninflammatory [100]. Space filling, cell coating, structural stabilization, cell protection, and wound healing are just a few of its numerous biological activities. Due to its inherent beneficial natural qualities, HA is used in biomedical, pharmacological, and aesthetic applications [100,101,102]. The abundance of carboxylic and hydroxyl acid groups promotes efficiency. Using conjugation, chemical bonding, and crosslinking, these functional groups can aid in the creation of new functional groups. Using a functional crosslinker, it is possible to create the useful microgel or nanogel from HA biopolymers in a straightforward manner [99,100,101,103].

**Figure 6:**
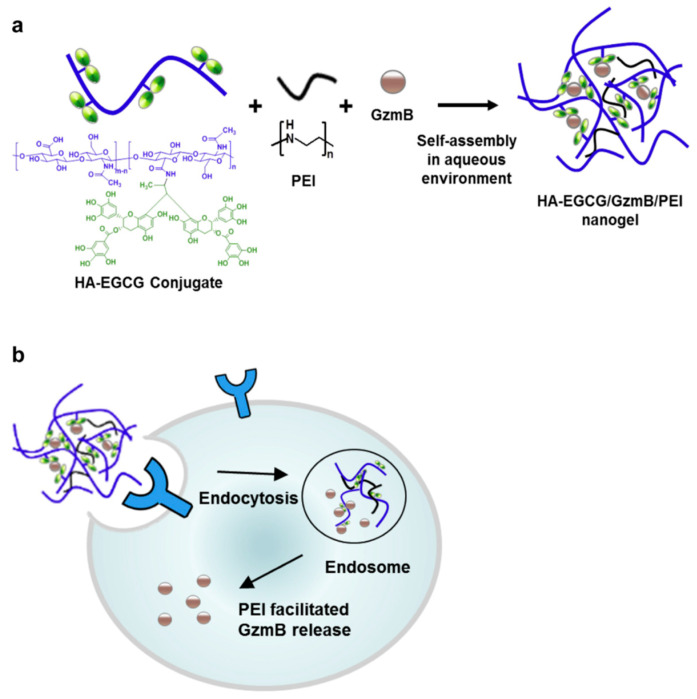
(**a**) Creation of HA-EGCG, PEI, and GzmB-containing self-assembled nanogels and (**b**) nanogel uptake via CD44-mediated mechanisms and PEI’s stimulation of GzmB release from endosomes result in the death of cancer cells [104] (reuse permitted by Elsevier).

In an effort to specifically transport granzyme B (GzmB) into cancer cells, Liang et al. developed a unique ternary nanogel based on the self-assembly of hyaluronic acid–epigallocatechin gallate conjugates (HA-EGCG), linear polyethylenimine (PEI), and GzmB (Figure 6) [104].

Studies on lysozyme activity and fluorescence quenching showed that EGCG moieties enhanced protein binding through physical interactions, which produced robust nanogels. An important cytotoxic response was seen in vitro when GzmB-encapsulated HA-EGCG nanogels were used to treat CD44-overexpressing HCT-116 colon cancer cells. Studies on intracellular trafficking and caspase tests confirmed the result that GzmB administration to the cytoplasm of the cells caused apoptosis, which caused the cellular death. When CD44-deficient cells were treated with HA-EGCG nanogels that were GzmB-encapsulated, minimal cytotoxic effect was seen. The potential use of HA-EGCG as efficient intracellular protein carriers for specific cancer therapy is underlined by this study.

Advanced boron neutron capture therapy (BNCT), an efficient radiation therapy for invading malignant tumors, has a lot of potential for boron-rich nanocarriers. They may also be used for dosage calculation and image-guided BNCT to increase the effectiveness of tumor treatment if they can be observed noninvasively and in real-time to measure local boron concentration. In order to overcome this difficulty, a brand new study outlines the invention of a theranostic nanogel that is rich in 10B and fluorescent dye, allowing for selective imaging and adequate boron deposition at the tumor location [105]. Through the simple process of temperature-triggered assembly of hyaluronic acid modified with a thermoresponsive terpolymer, boron-rich and fluorescent nanogels can be produced. The potential of theranostic hyaluronic acid nanogel as a boron delivery mechanism for the application of BNCT in brain cancer and sarcoma was suggested by this study.

**Figure 7:**
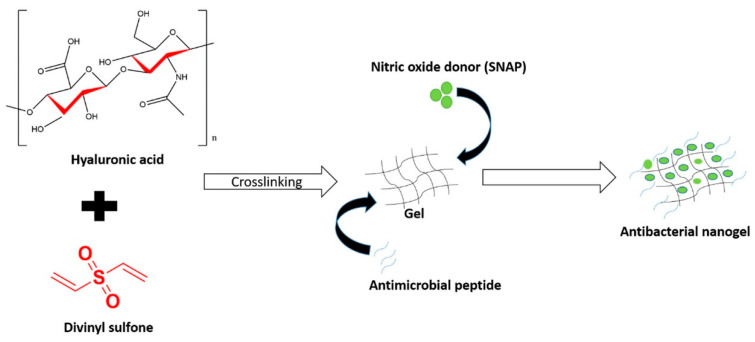
Crosslinking reaction and incorporation of the AMP and NO donor (SNAP) into the nanogel lead to the creation of the antibacterial nanogel [106] (reuse permitted by Elsevier).

Because they are linked to recurring and chronic infections as well as antibiotic resistance, biofilms are a global health concern. To combat bacteria and biofilms, Fasiku et al. prepared a nanogel for the simultaneous delivery of nitric oxide (NO) and antimicrobial peptide (AMP) [106]. Hyaluronic acid solution was crosslinked with divinyl sulfone to create the NO-releasing nanogel, which was then thoroughly described (Figure 7). 

The nanogel was demonstrated to be biocompatible, injectable, and as sustaining NO release over a 24 h period. The NO/AMP-loaded nanogel revealed a broad spectrum antibacterial/antibiofilm action in in vitro antibacterial experiments. With MIC values of 1.56, 0.78, and 0.39 g/mL against Escherichia coli, methicillin-resistant Staphylococcus aureus, and Pseudomonas aeruginosa bacteria, respectively, the NO-releasing nanogel showed a stronger antibacterial impact than NO alone. According to the antibiofilm results, nanogel loaded with AMP/NO reduced MRSA and P. aeruginosa biofilms by 12.5 and 24 times, respectively, when compared to nanogel loaded with only NO, whereas nanogel loaded with only NO reduced MRSA and *P. aeruginosa* biofilms by 7.0 and 9.4 times, respectively. The AMP/NO-releasing nanogel demonstrated the ability to inhibit bacterial infections as well as biofilms.

- Alginate

Alginate, a well-known linear anion polyelectrolyte polysaccharide made up of β-d-mannuronic acid (M units) and α-l-guluronic acid (G units), has been extensively employed as a synthetic hydrogel for the artificial extracellular matrix (ECM) [107]. Blocks of repetitive M residues (MM blocks), blocks of repetitive G residues (GG blocks), or blocks of hybrid M and G residues can be used to organize the monosaccharide repeats of alginate (MG blocks). Alginates with a higher proportion of G blocks produce gels that are noticeably stronger than alginates with a higher proportion of M blocks. This is because G residues have a higher affinity for divalent ions than M residues [108]. As a result, the M/G ratio and the arrangement of monosaccharide repeats mostly affect the physiochemical characteristics of alginate [109]. Alginic acid or its derivatives start to build a polymeric network when counter ions are introduced, and this process leads to the delivery system known as hydrogel. Any sort of cationic species can be used to start an alginate reaction; however, researchers have discovered that reactions involving calcium chloride and alginate are the most efficient and preferred [36].

In the research performed by Valentino et al., ionotropic gelation was used to create micro/nanogels through the interaction of cationic spermidine (SP) and anionic alginate [110]. The formulation with 0.17% (*w/w*) low viscous alginate and 0.017% (*w/w*) SP was chosen as the best sample based on the results. The development of nanogels was further verified by profilometric and FT-IR analyses that were conducted on this sample. Trehalose was shown to be necessary as a cryoprotectant agent to maintain the characteristics of nanogels during the freeze-drying process. Ultimately, the in vitro research on Schwann cells verified the formulation’s biocompatibility as well as its anti-inflammatory and antioxidant properties. The use of SP as a neuroprotective agent as well as a crosslinker agent is key to the work’s novelty. In addition to reducing oxidative stress and regulating the inflammatory state at the injury site, SP’s crosslinking action ensures the interaction with alginate and, as a result, the formation of micro/nanogels that, due to their composition and micro/nanoscale polymeric structure, provide a biomimetic environment. As a result, the produced nanogels are promising formulations that can be utilized to close the gap left following nerve injury. These nanosystems are highly desirable for tissue repair due to their network-like architecture, which is remarkably comparable to that of natural neural tissue, as well as their antioxidant and anti-inflammatory capabilities.

- Pullulan

Pullulan is nonionic, the unbranched, nontoxic, biodegradable, and water-soluble polysaccharide that is synthesized from starch by the fungus-like yeast named black yeast, also known as *Pullularia pullulans* or *Aureobasidium pullulans* [111]. Pullulan is a homopolysaccharide that is linearly polymerized by -1,6-linkages and consists of maltotriose units with three monosaccharides in each of its repeating units of 1,4-linked glucose molecules [59,112].

Pullulan has been extensively studied because of the modifications to functional derivatives that alter its properties and cause applied changes [113]. Hydrophobes such as cholesterol modify the pullulan polymer, making it behave like amphiphilic molecules that could serve as effective nanohydrogel carriers with amphiphilic characteristics [114]. Nanohydrogels made of pullulan were frequently used both in vivo and in vitro [115].

Therapeutic cancer vaccines must deliver vaccine antigens to macrophages and dendritic cells, which are antigen-presenting cells, in the lymphoid organs (spleen and lymph nodes), at the proper time in order to effectively trigger an antitumor immune response. For this reason, Muraoka et al. created a unique cancer vaccine that can, for the first time, deliver antigens for clinical cancer immunotherapy using self-assembled polysaccharide nanogel of pullulan with cholesteryl groups (CHP) [116]. In addition, they introduced a cutting-edge method that enhances the tumor microenvironment by controlling the activity of tumor-associated macrophages utilizing CHP nanogels. In combination with other immunotherapies, the change in macrophage activity by CHP nanogels had a strong inhibitory effect on cancers that were resistant to immune checkpoint-inhibiting treatment.

**Figure 8:**
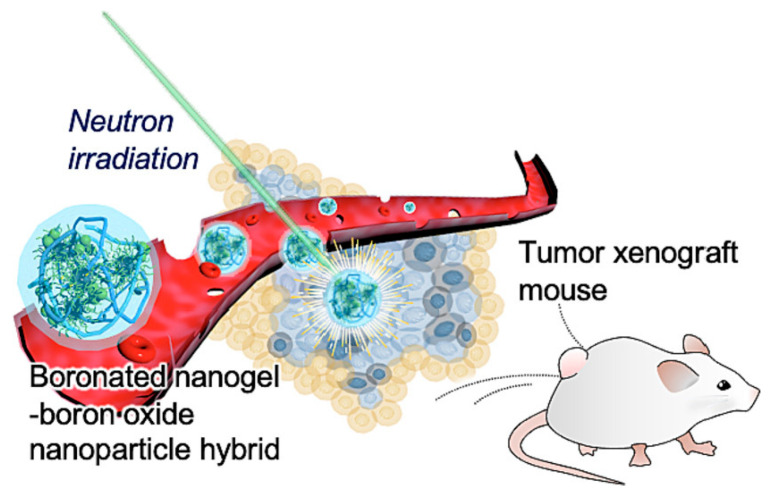
A hybrid nanoparticle that combines hydrophobized boron oxide nanoparticles with pullulan nanogel containing carborane was produced for boron neutron capture treatment [117].

Although boron neutron capture treatment is a promising method of treating cancer, it is difficult to distribute boron medicines. Kawasaki et al. developed a hybrid nanoparticle that combines a carborane-bearing pullulan nanogel with hydrophobized boron oxide nanoparticles (HBNGs), allowing them to create boron agents with a high concentration for effective distribution (Figure 8) [117]. By increasing the accumulation and retention amount of the boron agent within cells in vitro; on Colon26 cells, the HBNGs demonstrated greater anticancer activities than a therapeutic boron drug, L-BPA/fructose complex. Due to the improved permeability and retention effect, HBNGs accumulated in tumors, which made it possible to distribute boron drugs with great tumor selectivity to fulfill clinical needs. When intravenous boron neutron capture therapy was used, the tumor volume decreased without significantly affecting body weight, and three months after complete regression, no signs of tumor development were seen. Compared to L-BPA/fructose complex, HBNGs had higher therapeutic effectiveness. Boron neutron capture therapy utilizing HBNGs is a potential cancer treatment approach.

- Chondroitin sulfate

Chondroitin, a sulfated glycosaminoglycan, contains the residues N-acetyl-D-galactosamine and D-glucuronic [115]. It is found in many animals and even certain microorganisms [118]. Chondroitin sulfate has a wide range of bioactivities, including anti-inflammation, antiapoptotic, antioxidants, anticoagulation, and others. It is one of the primary components of the extracellular matrix (ECM) in many connective tissues, including skin, tendons, cartilage, bone, and ligaments [59,119]. The use of chondroitin sulfate as a first-line treatment for tissue engineering applications and osteoarthritis has been researched [120,121]. Additionally, chondroitin sulfate has been applied for protein delivery, anticancer drug targeting, and controlled release drug delivery [122]. Chondroitin-based nanohydrogels have grown in popularity in constructive processes such as various natural polymer assemblies or copolymer assembly in order to better understand how chondroitin sulfate contributes to the delivery of efficient treatments [115]. Tayeferad et al. created chondroitin sulfate–nisin nanogels (CS-N NGs) through electrostatic interaction to deliver nisin as an antibacterial agent used to treat bacterial infections induced by a few clinical strains of *Staphylococcus aureus* (*S. aureus*), both methicillin-sensitive and methicillin-resistant [123]. The produced CS-N NGs, with an average diameter of around 65 nm, were tested for stability using zeta potential measurements. The CS-N NGs became pH- and enzyme- responsive because of the existence of susceptible bonds in chondroitin sulfate, which resulted in controlled and efficient release of nisin in the simulated infectious medium. Additionally, the broth microdilution method was used to confirm the generated CS-N NGs’ capacity to eradicate a clinical methicillin-resistant *S. aureus* strain, and the cytotoxicity was evaluated using the MTT assay method on human dermal fibroblast cells. The results show that this adaptable drug delivery system is capable of effectively delivering natural antibiotics called cationic antimicrobial peptides for preventing the growth of methicillin-resistant *S. aureus* strains and treating methicillin-resistant *S. aureus* strain-induced subcutaneous infections by further destroying the pathogen.

Nanogel-based delivery systems have been widely used to treat cancer. In a study, chondroitin sulfate was grafted with octadecylamine using three distinct mole ratios (10, 20, and 30), and the resulting compounds were named CS–ODA1, 2, and 3, respectively [124]. Monodisperse nanogels with an average size of 63.08 ± 13.02 nm in dried state were produced via the self-assembly of CS–ODA conjugates having low critical concentrations of aggregation in aqueous solution. Due to their amazing capacity to swell under physiological conditions, these nanogels have a great possibility to avoid immunological reactions. Additionally, compared to free curcumin, they were able to extend the drug’s release by nearly 70 h and increase the cellular absorption of curcumin into the cytoplasm of cancer cells. According to the cytotoxicity data, it was also established that curcumin-loaded nanogels significantly raised the number of cells in the sub-G1 phase and exhibited considerable cytotoxicity against the MCF-7 cell line in 24 h, whereas blank nanogels showed to be practically noncytotoxic. Curcumin-loaded nanogels are able to penetrate cancer cells more easily due to chondroitin sulfate’s affinity for CD44 receptors, which contributes to considerable cancer cell deaths. The general conclusion from these findings was that the developed nanoscaled drug delivery technique would be a good candidate for further cancer therapy research.

- Carrageenan

The hydrophilic biopolymer known as carrageenan is produced by removing α-(1,4)-3,6-anhydro-galactose and β-(1,3) sulfated ɒ-galactose remaining substances from the extracellular matrix of red edible seaweeds [69]. The use of carrageenan spread rapidly in biomedical and biotechnological applications such as drug delivery systems, wound healing treatments, tissue engineering, and other industries due to its unique properties, including its increased molecular weight, gelation ability, high viscosity, biocompatibility, and biodegradability [125]. Additionally, a wealth of research has shown that carrageenan has anticoagulant [126], antiviral [127], antioxidant [128], immunomodulatory [129], and anticancer properties [130]. Carrageenan microgels can be created by using covalent linking to chemically crosslink the polymer chains with various crosslinkers or noncovalent interactions including ionic or hydrophobic interactions. The gelling characteristics of noncovalent bonding depend on transition from coil to helix when cations, such as Ca^2+^, K^+^, and Na^+^ [131], are present and the gelation functions depends on the amounts, valencies, and kinds of cations in the salts [69,132]. Additionally, in the temperature range of 25 to 45 °C, the temperature-responsive swelling κ-carrageenan nanogels go through a gel-to-sol transition that involves the breaking up of the gel’s physical links [1]. Nanogels made of crosslinked κ-carrageenan with an average size of less than 100 nm were produced using a combination of reverse microemulsions and thermally induced gelation [133]. The amount of biopolymer present affected the size of the nanogels at a constant ratio of water to surfactant concentration. It was discovered that the nanogels were thermosensitive in the range of 37 to 45 °C, which is suitable for living cells, and they undergo reversible volume shifts in response to thermal stimuli. This creates the opportunity to investigate the use of these nanogels in smart medical treatments, such as thermosensitive drug carriers. It was shown that temperature influences the release rate when examining the sustained release of methylene blue from the nanogels in proof of concept testing.

- Cyclodextrins

Cyclodextrins (CDs) are biocompatible cyclic oligosaccharides having units of D-glucopyranose with a-1,4-glycosidic bonding [134] can be changed using primary and secondary hydroxyl group esterification and etherification processes, resulting in a variety of CD derivatives [135]. Derivatized CDs differ from original CDs in terms of stability, solubility, and affinity to guest molecules depending on the nature and kind of the substituent. The pharmaceutical and cosmetic sectors have recognized CDs as advantageous vesicles that may encapsulate a variety of hydrophobic therapeutic compounds in their cavity due to their precise structure [136]. Due to their nonimmunogenicity, low toxicity, and biocompatibility, many researchers have produced CD-based nanogels as the carrier of numerous pharmaceuticals [59,137,138,139]. Oktay et al. produced flurbiprofen (FB)-loaded CD-based nanogel formulations and evaluated their dermal application [140]. In this study, nanogels were generated using emulsification solvent evaporation and integrated with hydroxypropyl methyl cellulose gel. Zeta potential, particle size, and polydispersity index measurements of the FB-loaded nanogel were measured to be −31.9 ± 0.5 mV, 295.5 ± 7.5 nm, and 0.361 ± 0.128, respectively. Plastic flow behavior was observed in all gels. Nanogels had a 97.55% FB loading efficiency, while FB-loaded nanogel in HPMC gel had a 96.88% loading efficiency. FB-loaded nanogels in HPMC penetrated the most FB without Transcutol^®^. None of the formulations led to skin irritation and cellular infiltration on a histological level. In conclusion, results show that dermal administration of hydrophobic medicines using nanogel formulations is a promising strategy.

- Starch

A well-known, affordable, and ubiquitous natural storage polysaccharide in higher plants, starch is a key component of both human and animal diets [141]. Starch can be found primarily in the leaves of all green plants as well as in the seeds, fruits, roots, stems, and tubers of the majority of plants [142]. In terms of structure, starch is a homopolysaccharide made up of D-glucose units connected by glycosidic interactions in the form of amylose and amylopectin [143]. 

**Figure 9:**
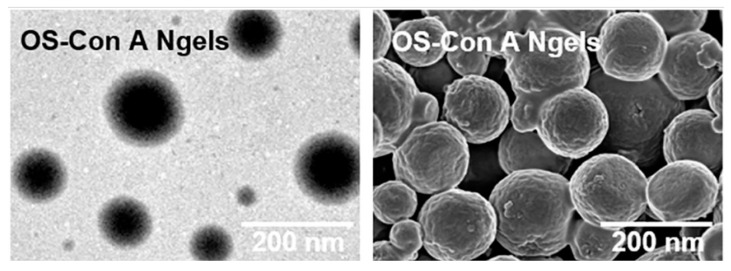
TEM and SEM images of the nanogels. Reprinted from [145]. Copyright (2022), with permission from Elsevier.

In addition to being a major food item, starch is currently used in nanomedicine including drug delivery systems due to its biocompatibility, biodegradability, and relatively easy isolation in pure form from their plant source [144]. In a study, a lectin Concanavalin A-modified oxidized starch nanogel was created by Bai et al. to dynamically control glucose in response to varying glucose concentrations [145]. In order to obtain maximum drug loading and glucose responsiveness, the Concanavalin A and glucose units on oxidized starch were doubly crosslinked to generate the nanogels. The nanogels were characterized using transmission electron microscopy (TEM) and scanning electron microscope (SEM) (Figure 9). The outcomes demonstrated that exenatide, an antidiabetic peptide medication, had a longer half-life and was released in response to high blood glucose levels in the presence of oxidized starch nanogels. It may efficiently absorb blood glucose while preserving glucose homeostasis. The nanogels also showed excellent biocompatibility in vivo and enhanced the plasma half-life and therapeutic effectiveness of exenatide. In summary, the nanogels served as a glucose-responsive platform with excellent potential for the delivery of peptide medications for the treatment of diabetes, preventing peptide pharmaceuticals from degrading in plasma.

- Cellulose

The most prevalent natural polymer on Earth is cellulose, which is widely dispersed in a wide range of sources, including plants (such as wood, cotton, or wheat straw), marine animals (such as tunicates), and bacterial sources such as algae (e.g., Valonia), fungi, and even amoeba (protozoa) [146]. A linear homopolysaccharide with anhydro-D-glucopyranose units connected by a β-(1 4)-glycosidic bond, cellulose is the basis for many biological materials. Because of its hydroxyl functional groups, cellulose has an inherent ability to self-assemble and create long networks through hydrogen linkages between individual molecules. Because of their low cost, wide availability, superior mechanical qualities, biodegradability, minimal cytotoxicity, and biocompatibility, cellulose and its derivatives are currently the subject of extensive investigation for potential use in medicine [147]. In the pharmaceutical industry, cellulose and its derivatives are well-known as “excipients” that are primarily employed to regulate the rate of drug release and obtain the proper drug concentration. As a result, numerous investigations on cellulose-based nanogels have been conducted, just as those on other polysaccharides have. As a result, cellulose-based nanogels have been the subject of several studies, similar to other polysaccharides.

In a recent study, by reducing silver nitrate with oxidized carboxymethyl cellulose (OCMC) in situ, functional and reactive nanosilver was developed [148]. Along with the reduction process, the OCMC polymer chain is also used to stabilize nanoparticles, creating a system where nanosilver remains trapped within OCMC gel. The TEM measurements reveal that the silver nanogels are around 22 nm. Both Gram-negative and Gram-positive bacteria were successfully eradicated by the nanogels’ strong antibacterial activity. A 0.3 mM concentration of silver nanogel was found to be effective in preventing bacterial growth. At a concentration of 0.4 mM Ag nanogels, 99.9% of *E. coli* and *S. aureus* were eliminated after 5 h thanks to the produced Ag nanogels’ antibacterial activity, which was dose-dependent. The bacterial cell wall was damaged by the nanogels, which also produced reactive oxygen species inside the cell, which led to cell death. Ultimately, the developed nanogels could be employed to create antibacterial implants and devices by coating polymer surfaces with them.

### 4.2. Protein-Based Nanogels

#### 4.2.1. Advantages of Protein Nanogels

Natural protein-based nanohydrogels are more desirable for use in biocompatible formulations than synthetic polymers because of their superior functional qualities, biocompatibility and biodegradability, good nutritional value, amphiphilic behavior, and lack of toxicities [149,150]. These nanohydrogels are superior to polysaccharide-based nanohydrogels due to the availability of more functional groups for modification (thiol, amino, hydroxyl, and carboxyl), more responsive delivery toward environmental stimuli, special recognition potential of some peptides, and self-assembling capabilities of certain peptides/proteins [151,152,153]. 

Besides that, the significant variation in natural polysaccharide-based hydrogels’ molecular weight, which necessitates sophisticated purification and analytical tools for evaluation, is one of the most frequent problems [154]. Proteins or peptides, on the other hand, often have a specific molecular weight, homogeneous physicochemical properties, and exhibit minimal batch-to-batch variance. Environmental factors such as pH, ionic concentration, and temperature have a significant impact on the physical properties of proteins, such as solubility, folding, and de-folding. For instance, the properties of peptides/proteins dramatically alter at the isoelectric point, which is used for the responsive, sensitive delivery of bioactive compounds [155,156].

Proteins and peptides are now recognized as a potent class of medicines [157,158]. However, using these biologics comes with a number of extra difficulties compared to using numerous traditional medications. The medicines’ poor stability and susceptibility to degradation present one difficulty [159,160]. Many medicines with peptide and protein origins affect intracellular targets [161,162]. Another difficulty is ensuring that medications made from proteins and peptides enter cells efficiently. It has been established that using nanosized formulations is a viable method for resolving some of these issues. Peptide and protein nanogels are especially promising because they make it possible to create nanosized formulations of these biologics with practically measurable encapsulation efficiency [14,163,164,165,166]. In the context of cancer immunotherapy, peptide/protein nanogels have also been investigated as carriers for the delivery of antigens and adjuvants [167]. Studies that have been published have employed both particular peptide epitopes and ovalbumin (OVA) (as a model antigen). Both noncovalent techniques that rely on electrostatic interactions [168,169] or host–guest interactions [170] and covalent crosslinking techniques have been used to create these peptide/protein nanogels [171,172,173]. Peptide/protein nanogels that include intermolecular disulfide bonds are particularly intriguing. Because of the disulfide crosslinks’ reduced sensitivity and cleavage upon exposure to the intracellular environment, these nanogels are appealing [166]. 

As a result of physical and noncovalent interactions, many short and ultrashort peptide sequences can spontaneously self-assemble into hydrogels, bypassing the need for crosslinking chemicals [174,175,176]. Fmoc-FF is one of the peptides that has been investigated the most. Ulijn et al. [177] and Gazit et al. [178] both identified it at the same time in 2006. Under physiological circumstances, this straightforward building block, utilizing a sheet motif, readily assembles into nanostructured fibrous hydrogels. This dipeptide and some of its analogs have been the subject of structural research, which has greatly increased our understanding of the self-assembling process [179,180,181,182]. Rosa et al. employed three alternative techniques—water/oil emulsion (W/O), top-down, and nanogelling in water—for creating stable peptide nanogels based on Fmoc-Phe-Phe-OH [156]. The impact of the formulation’s hydrophilic–lipophilic balance (HLB) on size and stability was also assessed. The anticancer agent doxorubicin, chosen as the model drug, was discovered to be encapsulated by the resultant nanogels with a drug loading comparable to those of the liposomes. For any clinical applications, nanogels with a diameter of about 200 nm are produced using top-down and W/O emulsion. The top-down methodology, as opposed to the W/O emulsion method, was thought to have a number of advantages. Firstly, using mineral oil during preparation is avoided when using the top-down approach. The extraction of the nanogel solution using organic solvents such as n-hexane can thus be avoided as well. Additionally, there are not many steps needed to prepare the top-down approach. The simple process along with excellent biocompatibility are beneficial characteristics from the standpoint of optimizing and enhancing their industrial manufacture. Additionally, this technique works well with techniques used to encapsulate anticancer medications. The next section discusses a few of the naturally occurring proteins that are widely used to make protein hydrogels.

#### 4.2.2. Natural Proteins Used in Nanogels

- Elastin

Nanogels are a novel class of drug delivery technologies that have better serum half-life and renal clearance. Nevertheless, synthetic polymeric nanogels are less biodegradable and immunogenic than others. Protein nanogels are gaining a lot of interest due to their non-immunogenicity; biodegradability; biocompatibility; and spatial, temporal, and mechanical adjustability.

The abundant structural protein elastin is very elastic and found in connective tissue’s extracellular matrix and the organs with the highest concentrations of elastin are the lungs, aorta, and skin. It affords the skin about a thousand times more flexibility than collagen protein [183]. The properties of elastin protein, such as its sensitivity to temperature and pH, non-immunogenicity, biocompatibility, biodegradability, inertness in the bloodstream, capacity for self-assembly, and capacity to cross the blood–brain barrier, make it a potential candidate for hydrogel production [107,108,184,185]. In previous research, the development of an elastin nanogel and its potential as a novel injectable nanodrug carrier were examined [185]. Elastin nanogel was produced using an inverse mini-emulsion method, and after being characterized and tested with five different prostate cancer cell lines of various origins, it was discovered to be cytocompatible and stable at room temperature. Elastin nanogel with rhodamine showed improved cellular absorption. Elastin nanogel complies with the requirements for injectable nanogels since it is compatible with vascular tissue as shown by hemolysis, blood smear, PT/APTT, C3a, and CBC complement activation assays. The developed elastin nanogel is efficient as a nanodrug injection carrier for cancer treatment. Additionally, hydrophobic drugs can be effectively delivered by encapsulating them in elastin nanogel.

- Collagen

An extracellular structural protein called collagen is mostly found in the connective tissues of a mammals and is responsible for the mechanical strength of the body. A range of 25 to 35% of the total protein composition is made up of collagen. The amino acids glycine, proline, and hydroxyproline are found in the collagen helix, commonly known as the triple helix [186]. Collagen is mainly produced by fibroblast cells and is mostly present in the ligaments, skin, and tendons. Collagen is extensively used in the fields of drug delivery, biomedical engineering, tissue engineering, and more recently, it has been used for the delivery of genes, nucleic acids, and proteins [108,187,188]. Pathan et al. effectively developed a curcumin nanoemulsion-loaded fish scale collagen (FSC)–hydroxypropyl methylcellulose nanogel (CNG) for use in dermatology [189]. In comparison to other formulations, research on ex vivo permeation of CGN showed extended release. In comparison to other formulations, the in vivo research of CNG showed a greater wound contraction value (100.42 ± 12.20%). The generated nanogel is safe for use in dermatology, according to a study on skin irritation. Studies on stability showed that the nanogel was stable. The created FSC–HPMC nanogel with curcumin nanoemulsion loaded is safe, has significant potential, and exhibits superior stability in applications for wound healing.

- Gelatin

Gelatin is created by partially hydrolyzing collagen and the peptides and proteins that make up gelatin are extracted from the bones, connective tissues, and skin. By denaturing or hydrolyzing collagen polymers, gelatin can be produced from the triple-helix, natural collagen polymers. Gelatin can absorb water between 5 and 10 times its weight [190,191]. Gelatin has gained popularity as a source of interest for the development of nanogels due to its availability and extensive commercial applications [115]. To this objective, fish gelatin methacryloyl (GelMA)-based nanogels were produced utilizing a water-in-oil nanoemulsion [192]. The properties and biocompatibility of fish GelMA nanogels were then assessed. The preparation process has an impact on the properties of the produced GelMA nanogels. We discovered that PBS nanogels/deionized water (D.W.) had high drug loading efficiency (77%), low polydispersity index (0.16), a desired particle size (200 nm), biocompatibility, the final dispersion solution of nanogels is distilled water, and the aqueous component of the mixture is phosphate-buffered saline. Studies on the drug’s in vitro release from doxorubicin–GelMA nanogels showed that it has a pH-dependent sustained release characteristic. The MTT assay was used to show that the released doxorubicin had an antitumor effect in NIH3T3 cells. For intracellular drug delivery, the increased release of doxorubicin under acidic conditions may be advantageous. Overall, it was confirmed that small-molecule substances can be delivered using fish GelMA nanogels without cytotoxicity or aggregation. It is worthwhile to conduct research on fish GelMA nanogel as a drug delivery system for a range of medications to treat various diseases.

- Silk Fibroin

The silkworm (Bombyx mori) is the main supplier and natural source of silk protein. Fibroin and sericin are the additional two proteins that make up silk. The proteins sericin and fibroin function cooperatively to build cocoons and are both bound together by a particular chemical interaction [193]. In addition to having many repeating units of the six amino acids Gly–Ser–Gly–Ala–Gly–Ala in its main structure, fibrin is made up of repetitive hydrophobic and hydrophilic residues [194,195]. Since silk has a rigid structure and a high tensile strength, the secondary structure of the fibroin protein shows an arrangement of clustered antiparallel sheets rich in glycine residue (around 45.9%) [194,196]. 

The ability of silk fibroin to form β-sheets enhances the mechanical performance of nano- and/or hydrogels. By stimulating the growth of β-sheets, certain biological features can be adjusted for their mechanical characteristics. For the induction of β-sheets confirmational alteration in silk fibroin structure to increase the workability of the resultant gel matrices, many physicochemical and enzymatic crosslinking techniques have been documented [193]. 

Increasing temperature and concentration have been observed to improve fibroin gelation. Moreover, the hydrophobicity of chains is reduced by the addition of a hydrophilic polymer or an increase in the acidity of the medium. This ultimately increases the water solubility and speeds up the gelation process [197]. 

**Figure 10:**
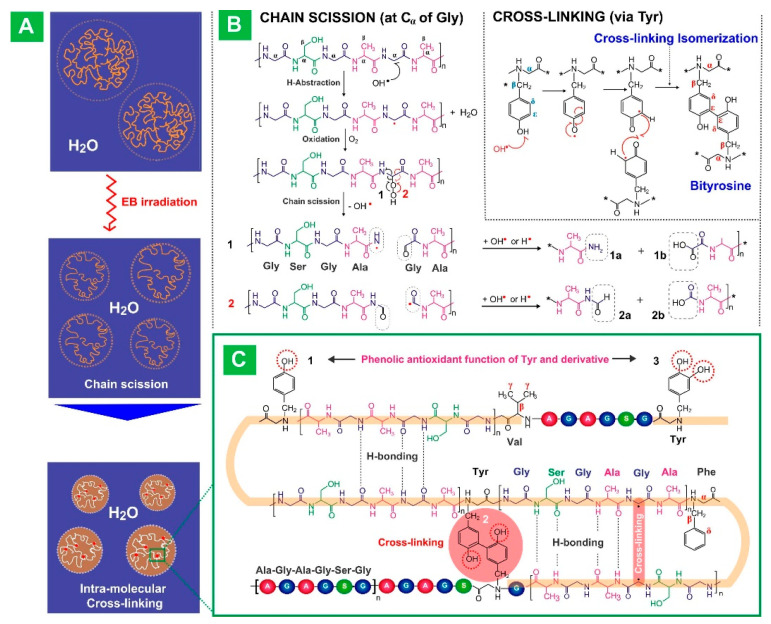
(**A**) A diagram illustrating the competitive chain scission and crosslinking mechanisms that radiation causes in aqueous SF to change it into colloids and micro-/nanogels; (**B**) hypothesized mechanism of a typical radiation-induced SF chain scission between a Gly unit and its neighboring amino acid and crosslinking by the creation of bityrosine bridges; and (**C**) simplified illustration of the entire SF structure with its phenolic side groups, which have antioxidant properties. Reprinted from [198]. Copyright (2022), with permission from Elsevier.

In order to fabricate micro- and nanogels with improved antioxidant capabilities, Soraya Wongkrongsak et al. investigated various aspects of the radiation-induced chemical modification of silk fibroin in pure water solution (Figure 10) [198]. 

Aqueous solutions made from samples of silk fibroin that had been exposed to radiation doses more than 20 kGy revealed the presence of nanogels with an average size less than 100 nm. Silk fibroin is mostly nontoxic, according to in vitro cytotoxicity experiments, and at specified concentrations (0.32 g/mL), silk fibroin formed in the form of micro- or nanogels after irradiation promotes the growth of keratinocyte cells. The radiation dose can be changed to produce particles of adjustable size while also greatly increasing antioxidant activity. This method is interesting in light of recent advancements in biomedical, therapeutic, and cosmeceutical applications because of how straightforward the treatment is and how well the additional qualities it produces balance out.

- Soy Protein

According to the research, one of the most essential nutrient, practical, and even health-promoting dietary proteins in human diets is soy protein [199]. It has been widely employed in the food and packaging industries because soy protein demonstrates a number of beneficial features, including the ability to gel, emulsify, absorb fat, and bind water [200,201]. Amphiphilic molding is possible using soy proteins. They serve as efficient nanocarriers for physiologically active compounds, particularly those with low solubility or low bioavailability, such as curcumin, by spontaneously combining with those molecules to create nanocomplexes [202]. In addition to their dual functioning as biologically active compounds and soy protein, soy protein nanocomplexes also show high solubility, high durability, delivery specificity, and excellent cell absorption [203]. Oil-in-water emulsions made of soy protein and polysaccharide can be stable (Yin et al. 2012). By utilizing a high-pressure homogenization to dissolve the original soy protein aggregates, nanogels made from these emulsions were loaded with folic acid (FA), which facilitates the binding of soy protein with soy polysaccharide and FA at pH 4 [204]. The soy protein was then subjected to a heat treatment that enabled it to gel, ensuring the creation of stable FA-loaded nanogels with a soy polysaccharide surface [135].

To obtain effective intracellular drug release and accumulation in A549 and A549/DDP cells, Cheng et al. created soybean protein-based nanogels with D-tocopheryl polyethylene glycol succinate (TPGS)-grafted and acid-responsive soybean proteins [205]. These smart nanogels had a uniform, globular structure and were approximately 200 nm in size. Cisplatin was successfully loaded into nanogels, and the cleavage of the ortho ester crosslinker enabled the in vitro drug release to exhibit an accelerated pattern at low pH. Additionally, a series of 2D and 3D cell studies confirmed the hypothesis that TPGS-modified nanogels could improve drug absorption and accumulation in drug-resistant cells, leading to greater anticancer effects. Moreover, the fact that the reversal multidrug resistance (MDR) mechanism of TPGS may produce mitochondrial depolarization and lower intracellular ATP level confirmed the mechanism. These findings clearly showed that the intelligent soybean protein-based nanogels had a tremendous potential to treat cancer cells more effectively and persistently, particularly when it came to overcoming solid tumors’ multidrug resistance.

## 5. Drug Release Mechanisms of Nanogels

Nanogels are primarily designed to achieve controlled medication release. Hence, achieving zero-order kinetics is the key goal. As nanogels are kinetically and thermodynamically stable, controlled release features are anticipated in these systems. Because of their better stability in biological fluids, nanogels release pharmaceuticals more gradually than micelles, which do so faster. In addition to polymer characteristics, the degree of crosslinking affects how the nanogels release their contents. Reduced crosslinking speeds up drug release since these systems are hydrophilic. The matrix’s mesh size significantly affects how long the release lasts, whether it occurs as a result of simple diffusion from the matrix system or stimulus-mediated release [206]. Basic release mechanisms of nanogels include (1) diffusion; (2) erosion of the nanogel matrices (3); ionic exchange with the environment; or (4) sensitivity to stimuli such as pH, temperature, magnetic field, light, and redox-response.

### 5.1. Diffusion

The simplest way to release drugs from nanogels is through diffusion, which has been exploited by several nanomedicines [36]. Because of the difference in concentration with the environment, the medication diffuses out of the gel. Inside the gel, where there is a higher concentration of the drug, it diffuses to a lower concentration (surrounding) [207].

### 5.2. Erosion of the Nanogel Matrices

A process known as nanogel degradation can also induce the release of drugs from molecules that have been encapsulated [36]. Nanogels’ biodegradable nature ensures decreased toxicity and eliminates unintended accumulation after repeated administration. The polymer backbone can be provided easily cleavable linkages. Certain reducing substances, pH, or even enzymatic activity might cause the breakdown. The rate of medication breakdown has been slowed down by encapsulation through hydrophobic contact [207]. 

### 5.3. Ionic Exchange with the Environment

Displacement with counterions is a different method of drug release. A negatively charged drug is exchanged for a negatively charged particle when a cationic nanogel containing the drug interacts with the negatively charged particles on the cell surface or surroundings [208]. 

### 5.4. Stimuli Responsiveness

Stimuli-responsive nanogels respond to changes in the physicochemical environment, including changes in pH, temperature, magnetic field, light intensity, and redox responsiveness. The release of therapeutic molecules is triggered by the current environmental stimuli. Nanogels that respond to stimuli are good candidates for systems that regulate drug release. Nevertheless, drug release in nonresponsive systems is based on water absorption, which has an unpredictable release pattern [207]. 

#### 5.4.1. pH-Sensitive Release

Due to the prevalence of pH gradients in both disease states and normal physiological systems, pH sensitivity has been extensively exploited in the design of different drug delivery devices for a long time. The pH levels of diseased tissues, such as infected, inflammatory, and cancerous tissue, are typically lower. Cancer tissues typically have an extracellular pH of 6.8 or perhaps much lower [209]. Similarly, for the normal systems, the gastrointestinal systems have a pH range that varies from 2.0 to 8.0, and this property has been successfully used in the development of pH-sensitive prodrugs and controlled release delivery systems [210]. 

Developing pH-sensitive nanogels is considerably simpler when using polymers with ionizable groups such as carboxylic acids and amines [211]. The majority of the interactions between the ionizable groups are pH-sensitive and can be used to create nanogels or conjugate with medicines for stable drug loading [212]. The most typical polymers in this method are polyelectrolytes, also known as ionomers. These polymers are composed structurally of carboxylic acid and/or amine functionality. These functionalities become ionized in response to any change in the pH of the surrounding environment, which alters the crosslinked structure of nanogels. Thermodynamic analysis of the polymer-solvent mixture and swelling of nanogels in their unionized form further confirm its mechanism, which is based on the elastic characteristic of polymers. Ionomers become ionized when dispersed in an aqueous medium with a certain pH and ionic strength, leading to electrostatic repulsions that explain the hydrogel network’s pH-dependent swelling/deswelling characteristics. The nature and properties of the polymers (such as concentration, ionic charge, degree of ionization, pKa value of ionizable group, and its hydrophilic/hydrophobic behavior) as well as the characteristics of the swelling medium, such as pH, ionic strength, and the type and charge density of counter ions, can be used to determine the extent of swelling of such polymers [212].

#### 5.4.2. Thermosensitive Triggered Release

Because they are made of thermosensitive polymers, temperature-triggered hydrogels can expand and shrink in response to changes in temperature [213]. According to their low critical solution temperature (LCST), temperature-triggered hydrogels can be divided into two groups: positive responsive and negative responsive. Positive temperature hydrogels shrink when the temperature is below LCST and expand when the temperature is above LCST. Hydrogels with negative temperature exhibit swelling at temperatures below LCST and shrinkage at temperatures above LCST [214]. Volume phase transition temperature (VPTT) is the range of temperature at which nanogels can alter their volume. The fluctuation in volume is typically used to improve encapsulation and regulate medication release [215,216]. One of the three mechanisms—simple diffusion, swelling, or degradation—is responsible for the release of thermosensitive nanogels. Depending on the nanogels’ composition, the release can either have a burst effect or not [207]. 

#### 5.4.3. Magnetic Field-Responsive Release

Iron oxide nanoparticles are combined with polymer to create nanogels that are specifically engineered to respond to magnetic fields. Since ferro- and ferrimagnetic nanoparticles have superparamagnetism capabilities, they are most frequently used in medication delivery [217,218]. Iron oxide nanoparticles that are nontoxic and biocompatible can be trapped in nanogels utilizing emulsion polymerization processes, making them suitable for medication administration. The drug distribution can be remotely managed by magnetic field-sensitive nanogels [217,219,220]. 

#### 5.4.4. Photo-Sensitive Release

Polymers with photoactive groups such as azobenzene, spirobenzopyran, or cinnamonyl comprise light-sensitive nanogels. The form and size of the nanogel change when these polymers are exposed to light because their double bonds change from trans to cis [221,222]. The hybrid nanogels, which combine polymer and noble metals, are another class of light-sensitive nanogels. In this instance, metals turn light energy into heat, causing a change in the polymer structure [223]. When exposed to certain radiations, photosensitive nanogels have the capacity for cistrans isomerization and can expand or shrink as a result of a change in temperature, which leads to the release of medicines [224].

#### 5.4.5. Redox-Responsive Release

Due to the presence of nicotinamide adenine dinucleotide phosphate (NADPH) and glutathione (GSH) reducing agents, tumors have a reducing environment. The tumor microenvironment is thought to have GSH levels four times higher than those of normal tissues, and this milieu promotes the rapid redox-responsive breakdown of nanocarrier systems, mostly by decreasing disulfide bonds [225]. Anticancer medications are released into the tumor’s oxidative state as a result of nanogel disintegration [226,227]. Moreover, the release primarily takes place in the target cells’ cytoplasm, which enhances the therapeutic benefits of anticancer medications [228,229].

## 6. Conclusions and Future Perspectives

Nanogels are used in a variety of medical applications including anesthesia, therapeutic drug carriers, sensors, wound dressings, diagnostic and imaging, and others. Their ability for architectural versatility makes it possible to incorporate inorganic nanoparticles, DNA, proteins, and a variety of other guest molecules [9]. Nanogels are remarkably versatile carriers for brain targeting due to their capacity to contain a wide range of moieties. It is possible to modify the size and properties of nanohydrogels to avoid phagocytic cell removal and both active and passive strategies can be used to reach the target [108].

The higher drug loading capacity, biocompatibility, swelling, and colloidal stability features of nanogels making them superior to alternative carrier systems. Nanogels have different unique characteristics depending on the kinds and sources of the monomer/polymer that make them up. The development of nanogels has shown to be an appealing research area for practical uses of polymeric compounds at the nanoscale. Nanogels have a wide range of applications and might offer alternatives in nanotechnology for overcoming cytotoxic effects [115].

Any biologically active substance can be placed into nanogels, which will then release the payload in response to both internal and external stimuli. The payload can be released immediately if there is a conformational change in the nanogels’ structure as a result of stimuli such as temperature, pH, glucose, enzyme, and redox potential. Additionally, external stimuli such as magnetic fields, ultrasound, and light may provide in the controlled release of drugs from the exterior [9]. The “intelligent nanogels” protect the drug molecule until the target cell internalizes it. Diverse crosslinking agents are crucial in decreasing the off-target effects because they administer the controlled release at the bioactive site and stimulus-sensitive drug delivery.

Nanogel synthesis improvements provide fine control over the size and shape that affect the biodistribution. The behavior of the nanogels when used in vivo can also be predicted thanks to advanced characterization methods.

The development of efficient and safe clinical treatments is the ultimate aim of research on drug delivery carriers. The production of nanogels with greater efficacy and less side effects is essential in order to improve therapies. In this regard, the capability of some nanogels to react to internal or external stimuli has drawn considerable interest over the past fifty years because they have the potential to be used in biomedical applications such as intracellular drug delivery systems and bioimaging.

The basis for the delivery of various therapeutic molecules for the treatment of diseases such as cancer [230], ischemia [231], HIV [232], and disorders of the central nervous system [233] using nanogels has been demonstrated by a number of studies with encouraging results. Nanogel surface functionalization with nanoparticles by various ligands has been shown to be effective in several targeting strategies, just like with others. Many articles show the extensive study of the creation of stimulus-responsive nanogels [234,235,236,237,238]. However, there is still a lot of work to be done before basic research can successfully be applied in clinical use. Even though nanogels have received prominence over the past decade, there are only a few cases of clinical research reported because of their obligatory careful engineering and complexity. In vivo studies are required to better comprehend how living organisms interact with nanogels. Although there has been a lot of study conducted, there is still some uncertainty regarding the pharmacokinetics and pharmacodynamics of these carriers, which must be clarified before clinical translation. Thus, more research is needed to understand their pharmacokinetic behavior, relations between nanogels and physiological conditions, biodistributions, and toxicities to successfully transform them into clinical delivery systems. In order to facilitate commercialization, crucial factors, such as therapeutic dose identification, regulatory concern setting, pharmacodynamics characterization, robust characterization procedures, optimization of storage conditions, homogeneity in the formulation, etc., must also be considered.

In conclusion, nanogels may become the next generation drug delivery systems and even start a revolution in this area due to their long term degradation and accumulation profile, careful engineering, thorough study of pharmacokinetics, and the realization that no delivery system is ideal regarding the benefits.

## Data Availability

Not applicable.
